# Efficacy of a Virtual Reality Biofeedback Game (DEEP) to Reduce Anxiety and Disruptive Classroom Behavior: Single-Case Study

**DOI:** 10.2196/16066

**Published:** 2020-03-24

**Authors:** Rineke Bossenbroek, Aniek Wols, Joanneke Weerdmeester, Anna Lichtwarck-Aschoff, Isabela Granic, Marieke M J W van Rooij

**Affiliations:** 1 Behavioural Science Institute Radboud University Nijmegen Netherlands

**Keywords:** anxiety, disruptive behavior, single-case study, applied game, serious games, special education, attention-deficit/hyperactivity disorder (ADHD), autism spectrum disorder (ASD), adolescents

## Abstract

**Background:**

Many adolescents in special education are affected by anxiety in addition to their behavioral problems. Anxiety leads to substantial long-term problems and may underlie disruptive behaviors in the classroom as a result of the individual’s inability to tolerate anxiety-provoking situations. Thus, interventions in special needs schools that help adolescents cope with anxiety and, in turn, diminish disruptive classroom behaviors are needed.

**Objective:**

This study aimed to evaluate the effect of a virtual reality biofeedback game, DEEP, on daily levels of state-anxiety and disruptive classroom behavior in a clinical sample. In addition, the study also aimed to examine the duration of the calm or relaxed state after playing DEEP.

**Methods:**

A total of 8 adolescents attending a special secondary school for students with behavioral and psychiatric problems participated in a single-case experimental ABAB study. Over a 4-week period, participants completed 6 DEEP sessions. In addition, momentary assessments (ie, 3 times a day) of self-reported state-anxiety and teacher-reported classroom behavior were collected throughout all A and B phases.

**Results:**

From analyzing the individual profiles, it was found that 6 participants showed reductions in anxiety, and 5 participants showed reductions in disruptive classroom behaviors after the introduction of DEEP. On a group level, results showed a small but significant reduction of anxiety (*d*=–0.29) and a small, nonsignificant reduction of disruptive classroom behavior (*d*=−0.16) on days when participants played DEEP. Moreover, it was found that the calm or relaxed state of participants after playing DEEP lasted for about 2 hours on average.

**Conclusions:**

This study demonstrates the potential of the game, DEEP, as an intervention for anxiety and disruptive classroom behavior in a special school setting. Future research is needed to fully optimize and personalize DEEP as an intervention for the heterogeneous special school population.

## Introduction

### Background

Adolescents attending schools for special education in the Netherlands (called “cluster 4 schools”) are characterized by profound behavioral and psychiatric problems [1], such as autism spectrum disorder (ASD), attention-deficit/hyperactivity disorder (ADHD), and oppositional defiant disorder. In addition, 27% to 32% of the Dutch heterogeneous special school population exhibits clinical levels of anxiety [2], indicated by high comorbidity rates between anxiety and ASD (40%) [3] and ADHD (25%) [4]. Anxiety symptoms significantly interfere with adolescents’ mental health, academic achievement, and social functioning [5]. Moreover, anxiety may underlie disruptive behaviors in the classroom, such as acting out spontaneously or expressing worries frequently, as a result of the individual’s inability to tolerate anxiety-provoking situations [6]. Individuals suffering from anxiety often have trouble regulating their behaviors in those situations because of underdeveloped self-regulation skills [7]. Clearly, interventions in special needs schools that help adolescents cope with anxiety and, in turn, diminish disruptive classroom behaviors are needed.

Conventional school-based interventions for anxiety are usually based on cognitive behavioral therapy (CBT) [8]. However, these conventional approaches pose limitations with regard to the special school population, as they often do not take the presence of comorbid disorders into consideration. For example, children with ASD may find it difficult to learn CBT skills such as cognitive restructuring because of their cognitive and social impairments [9]. Children with ADHD might also feel uncomfortable in talk therapies because higher-order abstractions may be challenging for them [10]. Moreover, conventional approaches entail challenges in their form of delivery because children are often not motivated to attend the didactic-based sessions nor to complete the homework assignments [11]. Within the school setting in particular, clinicians often fail to match interventions to the child’s readiness or motivation for change and hence struggle to keep students engaged [12,13]. Another limitation of CBT includes the use of decontextualized exercises that do not fully represent the authentic emotional and physical experiences associated with anxiety [14], limiting the transfer of the learned skills to other contexts (eg, the classroom setting). Finally, school clinicians have limited time to successfully deliver CBT [13,15]. As a result of these limitations and challenges, conventional school-based programs for anxiety often yield disappointing outcomes, with small-to-moderate effect sizes and intervention effects that do not sustain over time (see Mychailyszyn et al [16] and Werner-Seidler et al [17] for meta-analyses). Taking these limitations together, alternative interventions for anxiety need to be considered.

A promising alternative approach to enhance mental health among children and adolescents is the use of video games. A recent review showed that video games provide youths with immersive emotional experiences, teaching them new forms of emotional, cognitive, and behavioral strategies [14]. Video games may have the potential to address each of the limitations discussed. First, video games may be suitable for the heterogeneous special school population as they provide the player with visual aids and structured sensory information, an important prerequisite in treating anxiety in children with ASD [9,18]. Moreover, video games may impose less load on working memory capacities compared with the verbal reasoning required in CBT, which is an important requirement to treat anxiety in children with ADHD [19]. Second, video games hold great potential to intrinsically motivate and engage children, thereby addressing one of the most challenging tasks faced by (school) clinicians [20]. Third, video games provide youths with an opportunity to practice anxiety regulation skills until they are automatized and can be transferred to situations outside of the game [14]. Finally, a potential benefit of video games is their relatively low cost compared with traditionally delivered mental health interventions [21], thereby addressing the limited resources of clinicians in school settings. Overall, video games hold great promise as a novel intervention approach for adolescents with anxiety in the special school setting.

### DEEP

Recently, a virtual reality biofeedback game (DEEP) was developed as a potential intervention to reduce anxiety in youths [22]. In DEEP, players explore an underwater fantasy world by using their own breathing to control their movement. Players wear a belt with a stretch sensor around their waist just below their diaphragm. This sensor measures the expansion of the diaphragm associated with breathing, providing input to control in-game movement: the slower and deeper players breathe through their diaphragm, the better they can move around in the underwater world. The game aims to provide a relaxing and immersive experience for the player; there are no in-game goals to attain. DEEP targets a fundamental causal mechanism that contributes to the development and maintenance of anxiety symptoms: *physiological reactivity*. Anxious youths tend to experience hyperarousal (eg, rapid breathing or increased heart rate) in response to stressors [23]. The body’s physiological response interacts with negative cognitive biases (eg, focus on threat-relevant information [24]) to produce the negative affective states that characterize anxiety [25,26]. Thus, the ability to regulate physiological arousal may modify the interaction with negative cognitive biases, resulting in a positive change in the affective state.

The mechanism through which DEEP teaches players how to regulate physiological responses is diaphragmatic breathing [27]. Diaphragmatic breathing is the act of initiating breath into the lungs from contraction of the diaphragm muscle rather than the rib cage, which facilitates breathing efficiency and efficient exhalation [28,29]. Diaphragmatic breathing at a slow pace has been shown to evoke a relaxation response of the autonomic nervous system [30]. Hence, diaphragmatic breathing is commonly used as a relaxation technique in evidence-based treatments for anxiety [29,31].

**Figure 1:**
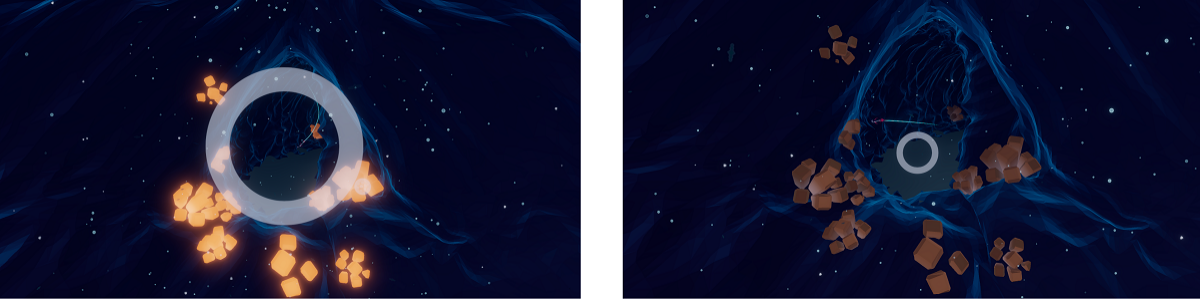
Visual circle that is depicted in the players’ visual field corresponding to an inhalation (left) and exhalation peak (right).

The breathing exercises incorporated in DEEP are based on biofeedback, which is defined as the process of feeding information back to the individual about one’s physiological state to gain awareness and control over physiological processes [32]. Biofeedback is an effective evidence-based therapeutic technique for regulating anxiety (see Schoenberg et al [33] and Weerdmeester et al [34] for recent reviews). In DEEP, biofeedback mechanics are applied in various ways. First, players receive feedback about their stage of breathing by their way of movement: when players inhale, an upward (when close to the ground) or a forward force is applied; when players exhale, these forces are strengthened. Second, players receive feedback about their breathing through visual cues. A circle is shown in the players’ visual field that expands with inhalation and contracts with exhalation (Figure 1). Finally, players’ breathing is mirrored by elements in the environment (eg, plants) that change in color, size, or movement accordingly. See Multimedia Appendix 1 for a short video of DEEP.

### Previous Research and Remaining Questions

Preliminary evidence for the efficacy of DEEP as an intervention for anxiety has been demonstrated in a recent pilot study [22]. In a sample of 86 typically developing children, DEEP reduced levels of state-anxiety directly after playing the game for 7 min. However, knowledge about the duration of the calm or relaxed state after playing DEEP is lacking, and it is also unknown if playing DEEP can reduce levels of state-anxiety in a clinical sample.

Next to the potential of DEEP as an intervention for anxiety, an additional yet uncertain beneficial effect of DEEP could be an improvement in the ability to regulate disruptive classroom behaviors. Previous research has shown that traditionally delivered deep-breathing exercises reduced rates of disruptive classroom behaviors (assessed by blind independent observers) among youths attending a special school for students with behavioral problems [35]. Moreover, a game-based intervention incorporating deep-breathing techniques and biofeedback has been found to significantly decrease self-reported externalizing behaviors of adolescents in residential youth care [36,37]. There may be two ways in which DEEP could potentially affect adolescents’ behaviors. First, playing DEEP may reduce disruptive classroom behaviors through its effect on anxiety levels. Disruptive behaviors of anxious youths are often driven by the individual’s inability to escape or avoid anxiety-provoking situations [6,38]. Not being able to withdraw from or avoid those situations (ie, blocking of the individual’s goals) may increase levels of frustration, which could result in subsequent externalizing behaviors [38,39]. Thus, if adolescents feel more relaxed and less anxious after playing DEEP, they may handle any subsequent anxiety-provoking situation better and feel less frustrated about being unable to escape. The diminished frustration may in turn make them less prone to show disruptive behaviors in the classroom.

Second, playing DEEP could potentially reduce disruptive behaviors through its effect on interoceptive awareness: the ability to recognize internal physiological states [40]. Playing DEEP might bring about increased interoceptive awareness as players are continuously informed about their breathing while playing. Heightened interoception might in turn enable adolescents to mobilize self-regulation resources as individuals who are able to focus on their body’s response to anxiety might recognize more quickly that control is needed [41]. Next to an efficient mobilization of self-regulation resources, previous research has shown that a better perception of one’s bodily signals may facilitate the use of adaptive emotion regulation strategies [42]. Those skills might empower adolescents to control their disruptive behaviors in the classroom.

### Design and Hypotheses

Our primary aim in this study was to investigate the effect of DEEP on daily levels of state-anxiety and disruptive classroom behavior in a clinical sample. It was expected that playing DEEP would reduce both participants’ state-anxiety and disruptive classroom behavior. Second, we explored the duration of the calm or relaxed state of participants after playing DEEP. No specific hypotheses were formed as this is the first study examining the duration of the effect of playing DEEP. To meet the research aims, we conducted a single-case experimental design (SCED) study in a special school setting. The SCED methodology was particularly appropriate because it allowed us to evaluate the efficacy of an intervention for individuals with heterogeneous characteristics (as is the case in special education [43,44]). This study followed an ABAB design, in which participants’ state-anxiety and disruptive classroom behavior in the absence of the intervention (A or baseline-withdrawal phase) were compared with participants’ state-anxiety and disruptive behavior during the intervention (B or intervention phase).

Disruptive classroom behavior outcomes were personalized per adolescent as difficulties to regulate oneself may manifest differently in each individual. This idiographic assessment approach may provide insight into whether the intervention is impacting the problems that teachers frequently observe and consider most important [45]. The Single-Case Reporting Guideline in Behavioural Interventions [46] was used to report this study.

## Methods

### Participants

Participants were 8 adolescents (mean age 14.67, SD 1.83 years) attending a secondary special school for students with behavioral and psychiatric problems in the northern part of the Netherlands. Adolescents were considered eligible for the study if teachers had the impression that the adolescent showed symptoms of anxiety, displayed disruptive behaviors in the classroom, and could handle the burden of completing momentary questionnaires (ie, 3 times a day). In total, teachers put forward 10 eligible adolescents. A school clinician contacted the parents of those 10 adolescents by phone to inform them about the study goals and to invite their child for participation. A total of 8 adolescents and their parents expressed their interest to participate in the study. The research team contacted these parents by phone to provide detailed information about the study’s procedure. All adolescents and their parents provided initial verbal consent and received an information and consent letter. Parental written informed consent was sent by mail. Individual demographic characteristics including participants’ age, gender, educational level, pretest trait-anxiety score, diagnoses, current medication, and treatment are provided in Table 1. All participants were of Dutch descent.

**Table 1:** Individualized description of participants’ demographics at pretest.

Participant	Age (years)	Gender	Educational level	Trait-anxiety scores^a^	Diagnoses^b^	Current medication	Current treatment
1	12.94	Male	Lower secondary vocational education	44	ADHD^c^ and ASD^d^	Methylphenidate	None
2	12.91	Male	Lower secondary vocational education	31	ADHD and ASD	Atomoxetine	None
3	13.90	Male	Lower and higher secondary vocation	37	ADHD and ASD	Methylphenidate	Psychotherapy
4	13.03	Male	Lower and higher secondary vocation	22	RAD^e^, ODD^f^, and ADHD	None	Psychomotor therapy
5	16.48	Male	Middle level vocational education	47	ASD	None	None
6	17.34	Male	Lower secondary education	36	ASD	None	Ambulatory care offered by the school clinicians
7	16.52	Male	Lower secondary education	36	ADHD and ASD	Methylphenidate^g^	Psychotherapy
8	14.22	Female	Lower secondary education	27	ASD, PD^h^, SAD^i^, and ED^j^	None	None

^a^To gain a general impression of adolescents’ predisposition toward anxiety, the trait-scale of the State-Trait Anxiety Inventory for Children [47] was assessed at pretest. Trait-anxiety scores can range from 20 to 60, with higher scores reflecting higher levels of trait-anxiety. Previous research suggests that children with subclinical and clinical levels of anxiety score on average 32.8 (SD 7.2) and 35.8 (SD 8.1) on the trait-scale, respectively [48,49].

^b^Diagnoses were derived from the electronic school database by the school clinician.

^c^ADHD: attention-deficit/hyperactivity disorder.

^d^ASD: autism spectrum disorder.

^e^RAD: reactive attachment disorder.

^f^ODD: oppositional defiant disorder.

^g^Participant 7 stopped taking medication on day 18, 19, and 20 of the study.

^h^PD: personality disorder.

^i^SAD: social anxiety disorder.

^j^ED: eating disorder.

### Design

This study followed an ABAB withdrawal or reversal design, with baseline (A_0_), intervention (B), and withdrawal or no intervention (A_1_) phases. A and B phases were alternated several times over a period of 4 weeks (ie, 20 schooldays in total). All participants started with the A_0_ baseline phase, which lasted for 5 or 6 days. After the baseline phase, the first intervention period (ie, B phase) began. The B phases usually lasted for 1 day, in which participants completed 1 DEEP session in the morning. However, some participants played DEEP on 2 subsequent days because the planned DEEP sessions did not synchronize with the participants’ schedule. The intervention days (ie, B phases) were alternated with withdrawal days, in which participants did not play DEEP (ie, A_1_ phases). All participants completed 6 DEEP sessions throughout the study period, except for participant 8 who completed 5 DEEP sessions because of illness. Another exception to this design was participant 5, who was doing an internship outside of the school for 2 days a week. Therefore, he participated in the study 3 days a week for 8 subsequent weeks (ie, 24 schooldays in total).

Throughout all phases, state-anxiety and disruptive classroom behavior were assessed with paper-and-pencil questionnaires 3 times per day. Adolescents reported their state-anxiety around 10:00 AM, 12:00 PM, and 2:00 PM. However, during B phases, participants filled in the first state-anxiety questionnaire directly after the DEEP session (between 8:45 AM and 10:15 AM). Teachers reported what they had observed about participants’ disruptive classroom behavior in the past 2 hours around 10:20 AM, 12:25 PM, and 2:30 PM. During B phases, teachers reported what they had observed about participants’ behavior *after* participants came back from the DEEP session. Therefore, 4 assessments around 10:20 AM were missing because, in those cases, the participant finished the DEEP session around 10:15 AM.

Participant 4 was observed by 3 teachers: 1 teacher observed for 4 days a week, 1 teacher observed for 1 day a week, and 1 teacher observed for 2 days in total when one of the other 2 teachers was ill. Participants 6 and 7 were classmates and were also observed by 2 teachers: 1 teacher observed for 4 days a week, and the other observed for 1 day a week. The remaining participants were observed by a single teacher. Participants 1 and 2 were classmates, so their behavior was observed by the same teacher.

### Procedure

At the start of the study, interviews with the teachers were held to discuss specific disruptive classroom behaviors of each participant. On the basis of their input, a personalized questionnaire about each participant’s disruptive behavior was developed. Afterward, participants filled in questionnaires regarding their demographics and trait-anxiety at pretest. Then, participants completed the ABAB study that lasted for 4 weeks. The study procedure was followed twice; in the first block, participants 1 to 4 participated, and in the second block, participants 6 to 8 participated. Participant 5 participated in both blocks. After participation, participants and their teachers both received a monetary compensation of €25.00. A total of 2 teachers received a monetary compensation of €37.50 because they had observed 2 adolescents for 4 or 5 days a week. Ethical approval for this study was obtained from the Radboud University Ethics Committee Social Sciences (ECSW-2017-038R1).

### Measures

#### State-Anxiety

Adolescents’ state-anxiety was assessed with the 6-item short form of the state scale of the Dutch State-Trait Anxiety Inventory (STAI) [50,51]. The 6 items reflect how calm, tense, upset, relaxed, content, or worried one feels at the moment. To ensure that the questionnaire would match the cognitive abilities of the sample, the questionnaire was adapted to a visual analog scale, consisting of a line of 10.0 cm. State-anxiety scores could therefore range between 0.0 (eg, not tense) and 10.0 (very tense). Item 1, 4, and 5 were recoded, and a mean score across all items was calculated, with higher values indicating more anxious feelings. The state scale of the STAI has shown good reliability and acceptable validity among various populations, including adolescents ([52,53]; for short form: [51,54]), and internal consistency was good (Cronbach alpha=.78) in this study.

#### Disruptive Classroom Behavior

On the basis of the interviews with the teachers, 2 or 3 disruptive behaviors were defined per participant (Table 2). During the ABAB study, teachers indicated how often those behaviors occurred in the past 2 hours by rating the items on a 6-point Likert scale: 0=*never*, 1=*rarely*, 2=*sometimes*, 3=*often*, 4=*very often*, and 5=*almost always*. Items 1 and 2 from participant 8 were recoded as the desired effect for this participant would be an increase in behavior rather than a decrease.

**Table 2:** Disruptive classroom behaviors of each participant, indicated by their teacher.

Participant	Behavior 1	Behavior 2	Behavior 3
1	Clears throat or sniffs nose	Asks for confirmation^a^	Gets off the chair and walks out of the classroom
2	Talks (loudly) out of turn	Asks for confirmation^a^	Gets off the chair and walks around the classroom
3	Taps fingers on the table or chair leg	Plays with or pulls hair	Gets off the chair and walks around or out of the classroom
4	Talks out of turn	Gets off the chair and walks around the classroom	Asks for confirmation^a^
5	Looks around during an independent work hour	Talks or laughs with classmates during an independent work hour	Asks for confirmation^a^
6	Shouts or talks loudly	Talks out of turn	N/A^b^
7	Looks around during an independent work hour	Asks for confirmation^a^	N/A
8	Asks a question^c^	Makes contact with classmates^c^	N/A

^a^For example: “Am I doing this right?” and “What are we going to do now?”.

^b^N/A: not applicable, because the teachers mentioned only 2 disruptive classroom behaviors for these participants.

^c^The desired effect for participant 8 was an increase in behavior rather than a decrease.

### Intervention

Participants completed DEEP sessions in a separate room at school. Upon arrival, participants first sat in a turnaround desk chair after which the DEEP breathing belt was placed around the abdomen. The DEEP belt contains an Arduino-compatible FLORA wearable electronic platform [55] that opens Arduino software [56] to keep track of real-time belt values. Afterward, the HTC Vive VR headset was adjusted to fit each player, and headphones were plugged in for the games’ music. Before starting the game, participants were instructed that they could move in the game by breathing deeply through their belly and by turning around in their chair. Finally, DEEP was started on a laptop, and a timer was started to keep track of time. Participants could play DEEP for 15 min but were allowed to stop earlier if they wanted to. On average, participants played DEEP for 12.41 (SD 3.76) min.

### Analyses

Means and standard deviations of A_0_, B, and A_1_ phase assessments of state-anxiety were calculated for each participant. In addition, the percentage of data points exceeding the median (PEM [57]) was computed for each participant. These scores reflect the percentage of data points in the B phases below the median of the A_0_ baseline phase. Afterward, a recent review [58] was used to choose the most appropriate analyses for the state-anxiety data.

To gain a general impression of the data, a visual analysis was performed involving the examination of trend (for the A_0_ baseline phase only), variability, and level [59]. Visual analysis was necessary to assess if the data pattern corresponded to what is expected from a baseline (ie, no stable upward or downward trend) and effective intervention (ie, less variability in B phases compared with the A_0_ baseline phase; reduction of the level of anxiety over the course of the intervention [58]). To assess if baseline data showed no stable upward or downward trend, trend stability envelopes [60] were calculated for each participant via a Web-based calculator [61]. Lane and Gast [60] proposed that stable trends are present when at least 80% of the data points fall within the envelope defined by the split-middle trend line plus or minus 25% of the baseline median. To compare variability in the B phases with variability in the A_0_ baseline phase, the median absolute deviation (MAD) was computed for participants’ A_0_ baseline phase and the B phases. To calculate the MAD of the B phases, data points of all B phases were merged together for each participant. The MAD was calculated on the 6 STAI items instead of participants’ mean anxiety to optimally represent participants’ variability.

To assess changes in level, a visual analysis usually focuses on mean score differences between phases [59]. However, this strategy has some limitations. First, mean score differences between phases do not take the presence of potential carryover effects into consideration, which refers to the continued impact of an intervention on subsequent (A_1_) phases [62]. Especially when adolescents have played DEEP several times, the intervention may have an irreversible effect on adolescents’ breathing skills. Second, the ABAB design consisted of several relatively short phases that also differed in length, making mean score comparisons between phases questionable. As such, it was decided to examine if participants’ level of anxiety decreased over time, regardless of phase, thereby keeping the temporal structure of the data intact. Levels of anxiety were classified with recursive partitioning, which is a data-driven approach that uses regression trees to identify segments of a time-series (of at least two data points) that have a stable mean value [63]. Recursive partitioning was performed with the ts_levels function of the casnet package (version 0.1.3, developed by Hasselman [64]) in R [65].

After the visual analysis, nonoverlap of all pairs (NAP [66]) was calculated, which reflects the proportion of NAP comparisons across A and B phases. NAP was calculated as the area under the curve percentage from a receiver operating characteristic analysis in IBM SPSS Statistics 21. NAP ranges of 0 to 0.65 are considered as weak effects, 0.66 to 0.92 as medium effects, and greater than 0.92 as strong effects [66]. The first NAP analysis included all A and B phases. To account for potential carryover effects, a second NAP analysis was conducted comparing the data points in the A_0_ baseline phase only with the data points in the B phases.

Finally, the between-case standardized mean difference (BC-SMD [67]) was computed to give an overall quantitative summary of the magnitude of change from A to B phases on a group level. The BC-SMD is an effect size designed specifically for SCED studies and can be compared with effect sizes for between-group designs. Moreover, the BC-SMD accounts for repeated intraindividual assessments and between- and within-case variances and includes a correction for a small sample bias [68,69]. The BC-SMD assumes that the outcome measurement is normally distributed and that there are no clear baseline trends [70]. A total of 4 separate BC-SMD quantifications were obtained. First, an overall change in level from all A to B phases was computed. Afterward, to investigate the duration of the effect of each DEEP session, we obtained overall changes in level from A to B phases measured around 10:00 AM, 12:00 PM, and 2:00 PM separately. The BC-SMD was calculated via a Web-based calculator, developed by Pustejovsky (version 0.3.1 [71]). Restricted maximum likelihood estimation was used to generate the design-comparable model effect size as it is the most flexible estimation model [72]. A random effect for treatment level was specified, which permits the treatment effect to vary across cases. Effect sizes of 0.20, 0.50, or 0.80 were interpreted as small, medium, or large effect sizes, respectively [73].

The same procedure, as described earlier, was repeated for classroom behavior. As NAP scores revealed the same pattern of results for all observed behaviors within the majority of participants, it was decided to only report the results of behavior 1 of each participant. However, for participants 2 and 3, behavior 2 was reported, and for participant 5, behavior 3 was reported because these were the only behaviors that showed medium effects for these participants; all other behaviors of these participants showed no effect.

In terms of missingness, 133 (27.0%) out of the 492 state-anxiety assessments were missing for reasons such as practical lessons, national holidays, or illness. Furthermore, 161 (32.7%) out of the 492 disruptive classroom behavior assessments were missing because of the absence of the adolescent (eg, practical lessons in front of another teacher) or lack of time. We decided not to use imputation strategies, as these percentages of missing data may not affect the quality of statistical inferences [74]. Moreover, the BC-SMD is a robust analysis strategy as it accounts for missing observations [70].

## Results

### Anxiety

#### Descriptive Statistics

Table 3 presents means and SDs on A_0_, B, and A_1_ phase assessments of anxiety and PEM scores for each participant. Overall, participants reported a mean anxiety of 3.32 (SD 1.81) in the A_0_ baseline phase, 2.35 (SD 1.48) in the B phases, and 2.30 (SD 1.31) in the A_1_ phases. Thus, participants’ mean anxiety scores fell within one-third of the full range of the scale in all phases. The findings indicate that participants’ anxiety scores may have declined over the course of the intervention. The minor difference between the participant’s anxiety in A_1_ and B phases suggests that carryover effects may have occurred. PEM scores ranged between 61% (11/18) and 100% (17/17), indicating that most assessments of anxiety were lower on the days of DEEP sessions compared with the A_0_ baseline phase.

**Table 3:** Means and standard deviations of anxiety by phase and percentage of data points exceeding the median scores for each participant.

Participant	A_0_ baseline phase, mean (SD)	B phases, mean (SD)	A_1_ phases, mean (SD)	Data points exceeding the median, n (%)
1	5.49 (2.24)	3.32 (1.79)	3.55 (1.47)	11 (92)
2	1.95 (1.59)	1.19 (1.60)	0.91 (1.07)	12 (80)
3	3.17 (1.68)	1.52 (0.90)	1.79 (1.02)	17 (100)
4	1.98 (0.56)	1.68 (0.33)	2.21 (0.71)	14 (82)
5	4.11 (1.45)	2.75 (1.78)	1.95 (0.51)	12 (86)
6	2.91 (1.49)	2.52 (1.16)	3.45 (1.13)	11 (61)
7	3.30 (1.29)	2.62 (1.27)	2.40 (0.99)	14 (78)
8	3.73 (0.99)	3.58 (1.21)	4.00 (1.19)	10 (67)

#### Visual Analysis

**Figure 2:**
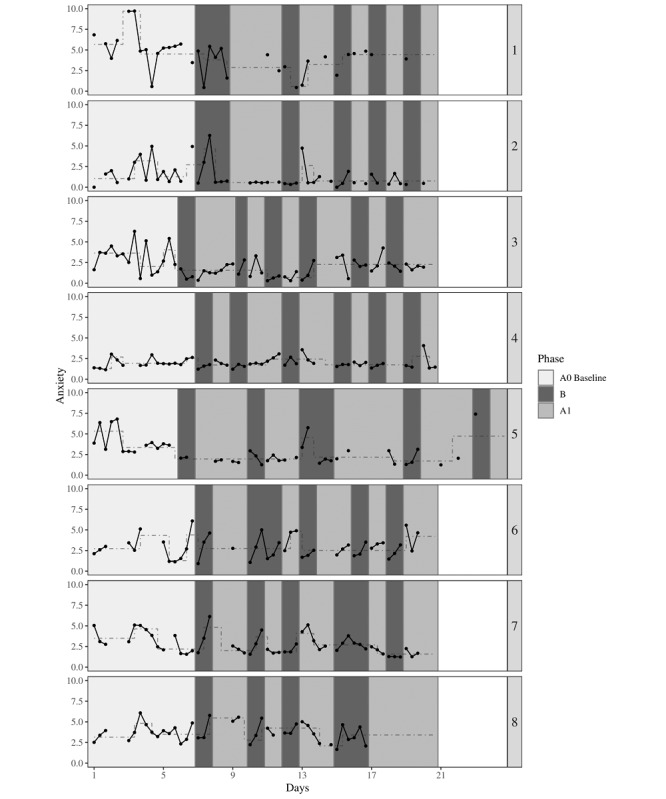
The effect of DEEP on anxiety for 8 adolescents. Every 3 data points represent 1 day (measured around 10:00 AM, 12:00 PM, and 2:00 PM). The dashed and stepped lines represent the relatively stable anxiety levels that were identified using recursive partitioning.

Each participant’s mean anxiety score is represented graphically in Figure 2. Trend stability envelopes revealed no stable upward or downward baseline trend for all participants (Table 4). The MADs showed that the variability in the B phases compared with the A_0_ baseline phase seemed to decrease for half of the participants (ie, participants 2, 3, 4, and 7; Table 4). In contrast, the data patterns of the other half (ie, participants 1, 5, 6, and 8) did not correspond to what is expected from an effective intervention as the variability in anxiety during the B phases compared with the A_0_ baseline phase increased or remained the same.

Finally, the changes in levels of anxiety that were identified by recursive partitioning are represented in Figure 2. Anxiety scores seemed to decrease for half of the participants (ie, participants 1, 3, 5, and 7) over the course of the intervention. However, scores of participant 7 should be interpreted cautiously as the decrease in anxiety seemed to correspond with the days he stopped taking his medication. A potential floor effect was detected for participant 2. This floor effect was considered as a desired effect as the levels of anxiety stabilized over the course of the intervention for this participant. No changes in anxiety level were identified for participants 4, 6, and 8 (Table 4).

**Table 4:** Results from visual analysis (baseline trend, variability, and change in level) and nonoverlap of all pairs on anxiety.

Participant	A_0_ baseline trend	Variability		Change in level	Comparison: A vs B		Comparison: A_0_ baseline vs B	
	Slope (% of data points within envelope)	MAD^a^ A_0_ phase	MAD B phases		NAP^b^	95% CI^c^	NAP	95% CI^c^
1	−0.11 (67)	3.41	3.85	Seems to decrease	0.72^d^	0.55-0.89	0.82^d^	0.66-0.98
2	0.04 (20)	0.59	0.30	Potential floor effect	0.63	0.45-0.82	0.74^d^	0.55-0.93
3	−0.16 (47)	2.82	1.04	Seems to decrease	0.66^d^	0.51-0.81	0.80^d^	0.64-0.96
4	0.03 (63)	2.97	1.63	No change in level	0.75^d^	0.61-0.88	0.68^d^	0.49-0.87
5	−0.21 (38)	1.48	1.48	Seems to decrease	0.58	0.39-0.77	0.81^d^	0.63-0.98
6	−0.11 (33)	1.85	2.00	No change in level	0.65	0.48-0.83	0.59	0.37-0.80
7	−0.35 (43)	2.00	1.63	Partly decreases	0.55	0.38-0.72	0.67^d^	0.48-0.86
8	−0.02 (60)	2.52	3.56	No change in level	0.58	0.39-0.77	0.56	0.35-0.77

^a^MAD: median absolute deviation.

^b^NAP: nonoverlap of all pairs.

^c^Confidence intervals are asymptotic.

^d^Medium effect.

#### Nonoverlap of All Pairs

NAP scores including all A and B phases are presented in Table 4. Participants 1, 3, and 4 showed changes in the medium range, indicating that their anxiety scores were lower during the B phases compared with the A phases. Regarding NAP scores including A_0_ baseline and B phases only participants 1, 2, 3, 4, 5, and 7 showed changes in the medium range. Carryover effects may have occurred for participants 2, 5, and 7 as they showed medium effects in the A_0_ baseline vs B phase comparison but no effect in the A vs B comparison.

#### Between-Case Standardized Mean Difference

The assumptions of normality and absence of clear baseline trends were tested and met. Therefore, the BC-SMD analysis was deemed appropriate. The overall A vs B comparison yielded an effect size of *d*=−0.29 (SE 0.11; 95% CI −0.51 to −0.08). This *d* statistic indicates a small but significant reduction of anxiety with the introduction of DEEP [73]. The A vs B comparison yielded a value of *d*=−0.43 (SE 0.15; 95% CI −0.74 to −0.15) for the data measured around 10:00 AM, a value of *d*=−0.34 (SE 0.17; 95% CI −0.68 to −0.02) for the data measured around 12:00 PM, and a value of *d*=−0.02 (SE 0.17; 95% CI −0.34 to 0.30) for the data measured around 2:00 PM. These *d* statistics indicate a medium significant reduction of anxiety directly after gameplay, a small but significant reduction 2 hours after gameplay, and no reduction 4 hours after gameplay (see Multimedia Appendix 2 for a graphical representation). Thus, the calm or relaxed state of participants after a DEEP session lasted for 2 hours on average.

### Disruptive Classroom Behavior

#### Descriptive Statistics

Table 5 presents means and SDs on A_0_, B, and A_1_ phase assessments of disruptive classroom behavior and PEM scores for each participant. Overall, participants reported a mean disruptive classroom behavior score of 2.60 (SD 1.72) in the A_0_ baseline phase, 2.11 (SD 1.53) in the B phases, and 2.21 (SD 1.49) in the A_1_ phases. These findings indicate a small decrease in participants’ disruptive classroom behavior during the intervention. The minor difference between participants’ classroom behavior in A_1_ and B phases suggests that carryover effects may have occurred. PEM scores ranged between 0% (0/11) and 77% (10/13), indicating that the effect of DEEP on classroom behavior varied between participants.

**Table 5:** Means and standard deviations of disruptive classroom behavior by phase and percentage of data points exceeding the median scores for each participant.

Participant	A_0_ baseline phase, mean (SD)	B phases, mean (SD)	A_1_ phases, mean (SD)	Data points exceeding the median, n (%)
1 - behavior 1	0.46 (0.66)	0.91 (1.14)	1.17 (1.64)	0 (0)
2 - behavior 2	2.23 (1.30)	1.38 (0.65)	1.06 (0.68)	7 (54)
3 - behavior 2	2.93 (0.83)	2.27 (0.96)	2.23 (0.75)	8 (53)
4 - behavior 1	3.00 (1.10)	1.31 (1.25)	2.50 (1.58)	10 (77)
5 - behavior 3	1.93 (0.62)	1.21 (0.80)	1.63 (0.50)	8 (57)
6 - behavior 1	2.00 (1.26)	2.38 (0.89)	2.14 (0.86)	2 (13)
7 - behavior 1	2.22 (1.20)	2.40 (1.30)	2.64 (0.50)	3 (20)
8 - behavior 1	6.00 (0.00)	5.27 (0.47)	5.78 (0.44)	8 (73)

#### Visual Analysis

**Figure 3:**
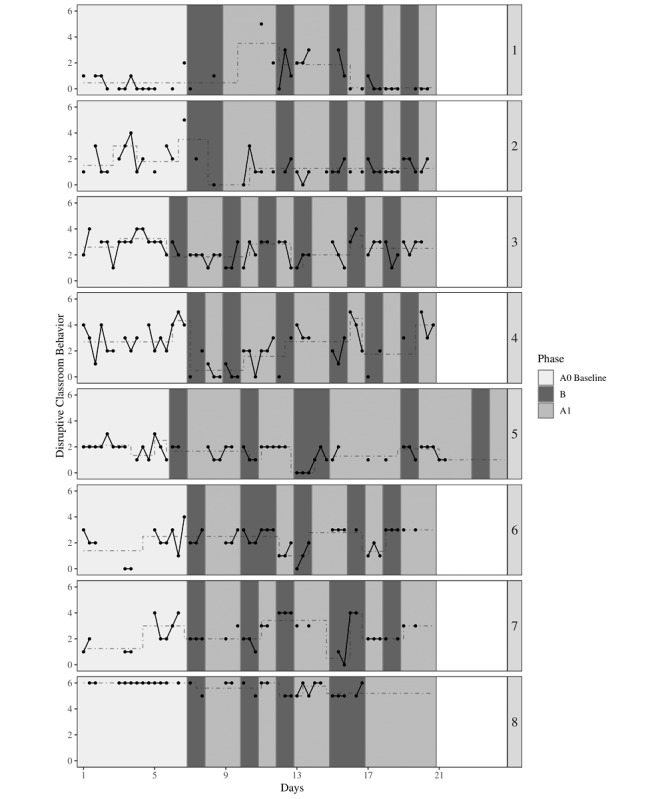
The effect of DEEP on disruptive classroom behavior for 8 adolescents. Every 3 data points represent 1 day (measured around 10:20 AM, 12:25 PM, and 2:30 PM). The dashed and stepped lines represent the relatively stable disruptive classroom behavior levels that were identified using recursive partitioning.

Each participants’ classroom behavior score is represented graphically in Figure 3. Trend stability envelopes revealed no stable upward or downward baseline trend for all participants (Table 6). The MADs showed that the variability in the B phases compared with the A_0_ baseline phase decreased only for participant 6. However, this finding should be interpreted cautiously as the level of disruptive behavior for this participant seemed to increase over the course of the intervention. The data pattern of all other participants did not correspond to what is expected from an effective intervention as variability in disruptive behaviors in the B phases compared with the A_0_ baseline phase increased or remained the same (Table 6).

Finally, the changes in relatively stable levels of disruptive classroom behavior that were identified by recursive partitioning are represented in Figure 3. Desired effects were found for half of the participants (ie, participants 2, 4, 5, and 8) as their disruptive behavior seemed to decrease over the course of the intervention. All other participants did not show any desirable effects in terms of their behavior (Table 6).

**Table 6:** Results from visual analysis (baseline trend, variability, and change in level) and nonoverlap of all pairs on disruptive classroom behavior.

Participant	A_0_ baseline trend	Variability		Change in level	Comparison: A vs B		Comparison: A_0_ baseline vs B	
	Slope (% of data points within envelope)	MAD^a^ A_0_ phase	MAD B phases		NAP^b^	95% CI^c^	NAP	95% CI^c^
1 - behavior 1	−0.07 (0)	0.00	1.48	Partly increases	0.45	0.24-0.65	0.40	0.16-0.63
2 - behavior 2	0.07 (38)	1.48	1.48	Seems to decrease	0.50	0.31-0.68	0.68^d^	0.47-0.89
3 - behavior 2	0.00 (57)	0.00	1.48	No change in level	0.57	0.40-0.75	0.69^d^	0.50-0.89
4 - behavior 1	0.12 (44)	1.48	1.48	Partly decreases	0.77^d^	0.62-0.91	0.83^d^	0.68-0.98
5 - behavior 3	0.00 (64)	0.00	1.48	Partly decreases	0.68^d^	0.50-0.86	0.73^d^	0.55-0.92
6 - behavior 1	0.00 (36)	1.48	0.00	Seems to increase	0.41	0.23-0.59	0.40	0.17-0.63
7 - behavior 1	0.30 (67)	1.48	1.48	No change in level	0.52	0.31-0.73	0.45	0.21-0.69
8 - behavior 1	0.00 (100)	0.00	0.00	Seems to decrease	0.82^d^	0.64-0.99	0.86^d^	0.70-1.00

^a^MAD: median absolute deviation.

^b^NAP: nonoverlap of all pairs.

^c^Confidence intervals are asymptotic.

^d^Medium effect.

#### Nonoverlap of All Pairs

NAP scores including all A and B phases are presented in Table 6. Participants 4, 5, and 8 showed changes in the medium range, indicating that their disruptive classroom behavior scores were lower during the B phases compared with the A phases. Regarding NAP scores including A_0_ baseline and B phases only, participants 2, 3, 4, 5, and 8 showed changes in the medium range. Carryover effects in terms of participants’ classroom behavior may have occurred for participants 2 and 3, as they showed medium effects in the A_0_ baseline vs B comparison but no effect in the A vs B comparison.

#### Between-Case Standardized Mean Difference

The assumptions of normality and absence of clear baseline trends were tested and met. The overall A vs B comparison of the BC-SMD analysis yielded a value of *d*=−0.16 (SE 0.12; 95% CI −0.39 to 0.05), indicative of a small, nonsignificant reduction of participants’ disruptive behavior with the introduction of DEEP [73].

## Discussion

### Principal Findings

This SCED study evaluated the efficacy of the virtual reality biofeedback game, DEEP, as an intervention to reduce anxiety and disruptive classroom behaviors in adolescents in a special school setting. The primary aim of the study was to test the effect of playing DEEP on daily levels of state-anxiety and disruptive classroom behavior. On a group level, results indicated a small-sized reduction of state-anxiety after the introduction of DEEP. On the individual level, strong evidence was found for 5 out of 8 participants as their NAP scores indicated a medium-sized reduction and their level of anxiety seemed to decrease over the course of the intervention. Moderate evidence was found for 1 participant, as NAP scores indicated a medium-sized reduction, but no change was found in the level of anxiety. In terms of disruptive classroom behavior, a small, nonsignificant reduction was found on the group level. On the individual level, strong evidence was found for 4 out of 8 participants as their NAP scores indicated a medium-sized reduction and their level of disruptive behavior seemed to decrease. Moderate evidence was found for 1 participant as the NAP score indicated a medium change, but no change was found in the level of disruptive behavior. The secondary aim of our study was to investigate the duration of the calm or relaxed state of participants after playing DEEP. Results indicated that, on average, the effect of playing DEEP lasted for 2 hours.

### The Effect of DEEP on State-Anxiety

In line with our hypothesis, the current findings indicate that, on a group level, DEEP reduces daily levels of state-anxiety. These results corroborate the previous pilot study conducted by Van Rooij et al [22], who found that playing DEEP for 7 min reduced levels of state-anxiety directly after the game. This study adds that DEEP also holds potential as an intervention for anxiety in adolescents with various disorders such as ADHD or ASD. Even though the direct effects of the evidence-based clinical techniques (ie, diaphragmatic breathing and biofeedback) incorporated in DEEP require further investigation, the techniques may have enabled individuals to gain awareness about and control over their diaphragmatic breathing, enabling them to regulate or dampen high arousal levels [27].

This study is the first study examining the duration of the effect of DEEP on state-anxiety and found that, on average, the calm or relaxed state of participants after playing DEEP lasted for 2 hours. This duration indicates that the effect of playing DEEP does not persist through the whole school day but may be particularly valuable to use in specific anxiety-provoking situations in class, such as during exams or when giving a speech. Although this study provides insight into the duration of the effect of DEEP on a group level, insight into individual variability in the duration of the effect is lacking. The NAP and visual analysis strategies used in this study are not suitable to analyze the duration of an effect on an individual level. It is possible that some individuals mainly reported reduced levels of state-anxiety directly after gameplay, whereas others still felt calmer or more relaxed at the end of a day playing DEEP. In addition, it is unknown if there are differences in the duration of the effect of DEEP between the 6 sessions. Participant 6, for example, showed a steep increase in anxiety during the day after the first and second DEEP session but seemed to feel calm or relaxed for a longer period after the last couple of DEEP sessions (Figure 2). Future studies could use more assessments per day and multilevel modeling techniques such as the N-of-1 randomized controlled trial methodology [75] to identify differences in the duration of the effect of DEEP between individuals and to investigate if the duration of the effect of DEEP might change over the course of the sessions within each individual.

### The Effect of DEEP on Disruptive Classroom Behavior

Analyses yielded mixed results for the efficacy of DEEP to reduce levels of disruptive classroom behavior. The results partly confirmed our hypothesis as 5 out of 8 individuals showed a reduction of disruptive classroom behavior after the introduction of DEEP. DEEP may have affected participants’ behavior in various ways. First, as high levels of anxiety may underlie escape-driven disruptive behaviors [6,38], participants in our study may have felt less anxious after playing DEEP and, as a result, were less prone to show disruptive behaviors in the classroom. Participant 5, for example, reported reduced levels of state-anxiety and was less likely to ask his teacher for constant confirmation on the days of DEEP sessions. Although findings need to be interpreted cautiously, participant 5 may have felt more relaxed after playing DEEP, enabling him to allow worrisome thoughts to pass through his mind (as hypothesized by Hayes et al [76]), which in turn may have led to a decreased need for constant confirmation from his teacher.

Second, DEEP may have reduced participants’ disruptive behavior through its effect on interoceptive awareness. Although empirical evidence is yet lacking, it is likely that participants’ interoceptive awareness improved over the course of the intervention because participants were continuously informed about their stage of breathing while playing DEEP. It has been theorized that increased interoceptive awareness may enable individuals to mobilize self-regulation resources [41], which are needed to regulate one’s behavior. Participants that benefited from DEEP in terms of their behavior may have been increasingly aware of their bodies and physiological responses, which may have enabled them to regulate their emotions and behavior in the classroom. To illustrate, after the intervention period, participant 8 noted that she learned to “...pay attention to my breathing more often, for example when I stress about an exam. It happened once, for an English exam. I tried to breathe in and out more deeply.” Supposedly, this participant became more aware of her bodily response to stress and tried to regulate heightened arousal levels by breathing. This self-regulation strategy may have helped her to cope with stressful situations in class, resulting in an increase in her participation (eg, asking questions to the teacher) in the classroom. Nevertheless, these explanations remain speculative; future studies are warranted to examine the underlying mechanisms (eg, interoceptive awareness and self-regulation) by which DEEP might affect disruptive behaviors.

Contrary to our hypothesis, we found a small, nonsignificant effect of DEEP on disruptive behavior on a group level. The 3 participants that did not seem to benefit from DEEP in terms of their behavior may have cancelled out the effect of the participants that did, leading to an unobservable effect on a group level. There are several possible explanations why DEEP did not affect the behaviors of those participants. First, anxiety may not have been the cause of disruptive behaviors in the classroom for these individuals. Rather, neurological deficits in individuals with ADHD or ASD that are associated with attention-related problems [77-79] may have caused the disruptive behaviors of the individuals that did not improve. Hence, DEEP may not have been targeting the underlying mechanisms responsible for the disruptive behaviors of these adolescents. Second, there may have been individual differences in the extent to which participants were actually engaging with the diaphragmatic breathing mechanic while playing DEEP. Some individuals may not have developed the skill to breathe through their diaphragm, limiting them in their ability to regulate their behaviors in the classroom. Future studies are encouraged to investigate if individual profiles in learning diaphragmatic breathing skills (as measured by in-game diaphragm expansions) might underlie changes in disruptive behavior [22].

### Strengths, Limitations, and Future Directions

A clear strength of this investigation is that the study was conducted in a special school setting, thereby addressing ecological validity issues relevant to the school setting. Another strength is that we used an SCED, which is a suitable design to test interventions in the heterogeneous special school population [43,44]. In addition, the study followed all external validity recommendations for SCED studies as described in the Risk of Bias in *N*-of-1 Trials (RoBiNT [80]).

Regarding the RoBiNt recommendations for internal validity, not all recommendations were carried out. First of all, we did not randomize the beginning of the study phases. We explicitly chose not to randomize the conditions because of constraints of the school setting (eg, scheduling conflicts) and characteristics of the target group (eg, in need of structure). In terms of sampling of the target behaviors, the required minimum of 3 data points in each study phase [80] was not always met because of missing data. Another limitation was that neither participants nor teachers were blinded to the study phases as the intervention required that participants leave the classroom to play DEEP in a separate room. The lack of blinding may have caused bias in teacher and child reports as they knew DEEP was supposed to reduce anxiety and disruptive classroom behaviors. In addition, inter-rater reliability measures of the target behaviors were not computed as no blinded assessors were used. The study was also limited because the behaviors of participants 4, 6, and 7 were assessed by multiple teachers, thereby reducing the reliability of those observations. Future studies may benefit from blinded assessors to reduce bias, and it is also recommended to tailor the units of measurement to the specific disruptive behaviors as some of the disruptive behaviors identified in this study would be better suited to rate or frequency measures rather than duration.

Although this study provided initial insight into the efficacy of DEEP in a special school setting, future research is needed to fully optimize DEEP as an intervention for this heterogeneous population. The intervention effects of DEEP could be optimized using a parametric analysis of the optimal amount of play time for a given individual [81]. Moreover, the active intervention elements of DEEP (eg, diaphragmatic breathing and biofeedback) that are necessary to produce behavior change should be determined in future studies. Although there are problems in terms of specificity when using physiological measures, adding physiological measures to detect changes in arousal during and after playing DEEP may also help uncover the potential working mechanism of DEEP. In addition, the conditions under which DEEP may be most successful for certain individuals could be established by replicating the current single-case study in new settings. Some individuals may benefit most from playing DEEP right before (eg, exams) or after (eg, quarrels) stressful events, whereas others may show optimal effects when they play DEEP at fixed times. Finally, larger group studies and replicated single-case research may yield variables that moderate intervention outcomes such as trait-anxiety scores, comorbid disorders, age, and gender, which could be used to personalize DEEP.

### Implications

The aforementioned limitations notwithstanding, this study has important implications for clinical practice. Although it should be noted that findings need to be interpreted cautiously in a study of this type, this study demonstrated the potential of DEEP as an intervention to reduce daily levels of state-anxiety and disruptive classroom behavior in a special school setting. The results implicate the clinical techniques of diaphragmatic breathing and biofeedback in treating individuals with anxiety. Moreover, the results demonstrate that an applied game incorporating those techniques can be used either in isolation or as an add-on to existing interventions in a clinical sample. On overage, our results showed that the calm or relaxed state of participants after playing DEEP lasted for 2 hours. Therefore, school clinicians are recommended to tailor implementation strategies of DEEP to the individual with different needs early or later in the day, considering individual variation in the duration of the effect of DEEP. The implementation of game-based interventions might be a promising avenue for the special school setting, as video games can be tailored to the diverse needs and learning paces of the heterogeneous special school population.

This study also has strong implications for future research on behavioral health interventions. This study demonstrated that there are individual differences in the extent to which an applied game to enhance mental health is effective. Therefore, we need to tailor behavioral interventions to the personal needs of different individuals. Although the randomized controlled trial may be the golden standard in behavioral health intervention research, this costly and time-invasive method is limited because of a lack of attention paid to individual differences [82,83]. We advocate the use of SCED research to optimize health interventions through ongoing tailoring and testing [81]. Single-case designs provide an excellent opportunity to dynamically and efficiently assess the most promising intervention elements through systematic replication of single-case experiments [81]. Moreover, single-case designs may enhance our understanding of the conditions under which interventions are most successful by replicating single-case studies in new settings, thereby also establishing its generality. Knowledge derived from a body of SCED research about certain demographic and diagnostic variables, generality, and the conditions that may influence intervention outcomes may eventually enable (school) clinicians to personalize behavioral health interventions to the unique characteristics of the individual.

## Appendix

Multimedia Appendix 1 DEEP trailer.

Multimedia Appendix 2 Participants’ mean anxiety scores measured around 10:00 AM, 12:00 PM, or 2:00 PM, split out by phases.

## Abbreviations

ADHD: attention-deficit/hyperactivity disorder
ASD: autism spectrum disorder
BC-SMD: between-case standardized mean difference
CBT: cognitive behavioral therapy
MAD: median absolute deviation
NAP: nonoverlap of all pairs
PEM: percentage of data points exceeding the median
RoBiNT: Risk of Bias in N-of-1 Trials
SCED: single-case experimental design
STAI: State-Trait Anxiety Inventory
