# Supplementary figures and images for: Efficacy of a Virtual Reality Biofeedback Game (DEEP) to Reduce Anxiety and Disruptive Classroom Behavior: Single-Case Study
Source: JMIR Ment Health. 2020 Mar 24;7(3):e16066. doi: 10.2196/16066 (PMC7139423; doi:10.2196/16066)

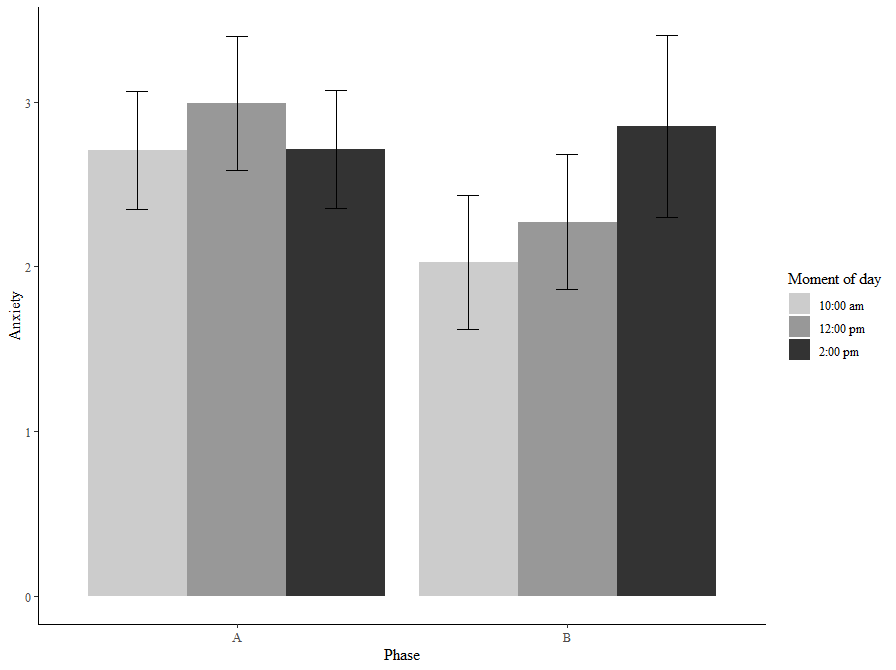

Supplement: Multimedia Appendix 2 [file mental_v7i3e16066_app2.png]
